# From victim to perpetrator to survivor: The psycho-social context of South African women offenders

**DOI:** 10.4102/sajpsychiatry.v24i0.1290

**Published:** 2018-10-03

**Authors:** Mohammed Nagdee, Lillian Artz, Carmen Corral-Bulnes, Aisling Heath, Ugasvaree Subramaney, Helena G. de Clercq, Helmut Erlacher, Carla Kotze, Gian Lippi, Samantha Naidoo, Funeka Sokudela

**Affiliations:** 1 Fort England Hospital, South Africa; 2 Department of Psychiatry, Walter Sisulu University, South Africa; 3 Department of Psychology, Rhodes University, South Africa; 4 Gender, Health and Justice Research Unit, University of Cape Town, South Africa; 5 Sterkfontein Hospital, South Africa; 6 Department of Psychiatry, University of the Witwatersrand, South Africa; 7 Valkenberg Hospital, South Africa; 8 Department of Psychiatry, University of Cape Town, South Africa; 9 Weskoppies Hospital, South Africa; 10 Department of Psychiatry, University of Pretoria, South Africa; 11 Forensic Mental Health Unit, University of Pretoria, South Africa

## Abstract

**Background:**

There is a paucity of research on women offenders in the South African context, particularly those referred for forensic psychiatric observation. Little is known about their life histories, the nature of their offences or the psycho-social contexts that enable, or are antecedents to, women’s criminal offending.

**Aim:**

This research study, the largest of its kind in South Africa, examined the psycho-social contexts within which women offenders referred for psychiatric evaluation come to commit offences. The profiles of both offenders and victims, as well as reasons for referral and forensic mental health outcomes, were investigated.

**Methods:**

A retrospective record review of 573 cases, spanning a 12-year review period, from 6 different forensic psychiatric units in South Africa, was conducted.

**Results:**

The findings describe a population of women offenders who come from backgrounds of socio-demographic and socio-economic adversity, with relatively high pre-offence incidences of being victims of abuse themselves, with significant levels of mental illness and alcohol abuse permeating life histories. The majority of index offences which led to court-ordered forensic evaluations were for violent offences against the person, with murder being the single most common index offence in the sample. Most victims of violence were known to the accused. There were also relatively high rates of psychotic and mood-spectrum disorders present, with relatively low rates of personality disorders. The majority of women were deemed to be trial competent and criminally responsible in relation to their index offences.

**Conclusion:**

It is recommended that more standardised and gender-sensitive forensic mental health assessment approaches, documentation and reporting be employed throughout the country. Future research should compare male and female offending patterns and forensic mental health profiles.

